# Routine Data Analysis of HIV Pre-Exposure Prophylaxis Use and Rates of Sexually Transmitted Infections Since Coverage of HIV Pre-Exposure Prophylaxis by the Statutory Health Insurance in Germany

**DOI:** 10.1007/s10508-024-02922-5

**Published:** 2024-08-06

**Authors:** Frederik Valbert, Daniel Schmidt, Christian Kollan, Patrik Dröge, Melanie Klein, Udo Schneider, Martin Friebe, Jürgen Wasem, Anja Neumann

**Affiliations:** 1 Institute for Healthcare Management and Research, University of Duisburg-Essen, Thea-Leymann-Str. 9, 45127 Essen, Germany; 2 Department of Infectious Disease Epidemiology, Robert Koch Institute, Berlin, Germany; 3 AOK Research Institute (WIdO), Berlin, Germany; 4 DAK Gesundheit, Hamburg, Germany; 5 Techniker Krankenkasse, Hamburg, Germany

**Keywords:** Human immunodeficiency virus, Pre-exposure prophylaxis, Sexually transmitted infections, Surveillance, Germany

## Abstract

Human immunodeficiency virus pre-exposure prophylaxis (PrEP) is considered as an effective protection against a human immunodeficiency virus (HIV) infection. However, it is still unclear, how PrEP use is associated with the incidence of sexually transmitted infections (STI) other than HIV. PrEP became reimbursable in Germany from September 1, 2019 for persons with statutory health insurance (SHI). With the EvE-PrEP study, the Federal Ministry of Health commissioned the evaluation of the effects of the new reimbursement situation in Germany. In the presented module of the EvE-PrEP study, routine data from three large German SHI funds were analyzed in anonymized form for the period January 1, 2019 to March 31, 2020. Data were analyzed regarding: Characteristics and adherence of PrEP users, treatment success of PrEP, and changes in STI incidence rates before and since PrEP use. The cooperating SHI funds collectively covered about 52% of the overall population in Germany in 2019. A total of 7102 persons with PrEP use were included into the analysis. These were predominantly male (99%), on average 37.4 years old and a high proportion of persons lived in large cities. The average quotient of PrEP daily defined doses and assumed days on PrEP was 87%. The average STI rates normalized per 100 person-years at individual level pre PrEP did not statistically significant differ compared to since PrEP (chlamydia: 17.5 vs. 17.6, gonococcal infection: 29.1 vs. 30.7, and syphilis: 14.6 vs.13.6). A large data set was used to evaluate the introduction of PrEP as a SHI benefit in Germany. A potentially suspected increase in bacterial STI incidence rates was not found. A rather high average adherence rate was observed. The very high proportion of men and people from the largest German cities among PrEP users is striking. These results could indicate barriers to PrEP access for people at risk of HIV, especially if they are women or people living in less urban areas.

## Introduction

Human immunodeficiency virus pre-exposure prophylaxis (PrEP) is considered as an effective protection against a human immunodeficiency virus (HIV) infection (Baeten et al., 2012; Fonner et al., 2016; Grant et al., 2010; McCormack et al., 2016; Molina et al., 2017; Spinner et al., 2016; Thigpen et al., 2012). There are also indications of the cost-effectiveness of PrEP, both internationally and specifically for Germany (Cambiano et al., 2018; Reitsema et al., 2020; van de Vijver et al., 2019). However, it is still unclear, which effects PrEP use has on the incidence of sexually transmitted infections (STI) other than HIV. Regarding this topic, published evidence does not show consistent results. Some of the studies report increases (e.g., due to changed risk behavior), others decreases or no change in incidence/prevalence. In further studies, rates of specific STIs are developing differently (Beymer et al., 2018; Fayaz Farkhad et al., 2021; Jenness et al., 2017; McCormack et al., 2016; Molina et al., 2017; Nguyen et al., 2018; Reitsema et al., 2020; Schmidt et al., 2023; Serpa et al., 2020; Streeck et al., 2022; Traeger et al., 2019; Werner et al., 2018, 2019). For example, Traeger et al. observed significant increases in syphilis, gonorrhea, and chlamydia among men who have sex with men or bisexual men using PrEP in Australia, even after adjusting for higher STI testing rates after PrEP initiation (Traeger et al., 2019). In contrast to these findings, Schmidt et al. reported significant decreases in the incidence rates of chlamydia, gonorrhea, and syphilis among PrEP users after PrEP initiation. However, SARS-CoV-2 pandemic prevention measures and behavioral changes in Germany are also mentioned as a possible explanation for these decreases (Schmidt et al., 2023). In a model by Reitsema et al., a reduction in gonorrhea transmissions was expected in a PrEP program for high-risk men who have sex with men due to frequent STI testing and treatment. For example, McCormack et al., Molina et al., and Streek et al. provided examples of studies that found no significant effect of PrEP on STIs. Serpa et al. observed that PrEP implementation was associated with increasing syphilis and gonorrhea rates and decreasing chlamydia rates. Serpa et al. (2020) pointed out that further research is needed to confirm and causally explain these associations. After all, PrEP programs appear to be associated with factors that can both potentially increase and potentially decrease STI rates. However, there is no consistent description of which factors dominate in practice.

In 2016, PrEP became available to self-pay patients in Germany (European Medicines Agency, 2016). However, patients had to pay not only for the pills, but also for medical and laboratory services related to PrEP use. Under these conditions, Marcus et al. (2022) estimated 1178 PrEP users in Germany in November 2017, 7440 in July 2018, and 8916 in May 2019. Due to the “Appointment Service and Care Act” (original title: “Terminservice- und Versorgungsgesetz”), PrEP became reimbursable in Germany from September 1, 2019 for all persons with statutory health insurance (SHI) from the age of 16 who have a substantial risk of HIV infection. This includes, for example, men who have sex with men or transgender people, with condomless anal sex, or who have had a STI in the last 12 months, intravenous drug users without sterile injection equipment, and HIV serodiscordant partners with detectable viral load. However, at the individual level, substantial risk can also be seen in less well-defined groups of people, for example, generally in people who have sex without a condom with a partner who is likely to have an undiagnosed HIV infection (Federal Ministry of Justice, 2019; National Association of Statutory Health Insurance Physicians, National Association of Statutory Health Insurance Funds, 2019). Now that PrEP, including the associated medical and laboratory services, is covered by the SHI system, a significant increase in the number of users can be expected (Marcus et al., 2022). Accordingly, the impact of PrEP, for example on the incidence of bacterial STIs, will become more relevant.

Health insurance is mandatory for all residents of Germany. Depending on characteristics such as income and previous insurance periods, people can choose between private health insurance funds and various SHI funds. The SHI takes a central role, as approximately 90% of Germans are covered by SHI. The insurance benefits are very broad and are based on a right to adequate, needs-based medical treatment in accordance with the generally accepted state of medical science. This includes medical, dental, and psychotherapeutic treatment, the provision of drugs, therapeutic products and treatments, home care, hospital treatment and medical rehabilitation services. SHI funds may also provide additional services on a voluntary basis. As part of the reimbursement process for drugs, medical devices, or medical services, data that includes billing codes, Anatomical Therapeutic Chemical Classification (ATC) codes or the 10th revision of the *International Statistical Classification of Diseases and Related Health Problems* (ICD-10) codes are submitted to insurance companies. These data, known as routine data, have become an established basis for research in Germany (Gansen, 2018; Schubert et al., 2008).

With the “Evaluation of the introduction of HIV pre-exposure prophylaxis as a benefit of the statutory health insurance system” (EvE-PrEP) study (original study name: “Evaluation der Einführung der HIV-Präexpositionsprophylaxe als Leistung der Gesetzlichen Krankenversicherung”), the Federal Ministry of Health commissioned the evaluation of the effects of the new reimbursement situation in Germany with regard to the number of users, the number of prescriptions, PrEP adherence, user behavior, PrEP treatment success, changes of STI rates other than HIV, and barriers to access PrEP as well as groups of people who would benefit from PrEP but are not yet reached. Various different methods and data bases were used for this purpose (Schmidt et al., 2021).

The presented routine data analysis is a module of the EvE-PrEP study led by the Robert Koch Institute (Schmidt et al., 2021). The aim of this sub-study within EvE-PrEP led by the Institute for Healthcare Management and Research of the University Duisburg-Essen was to estimate the number of PrEP users and describe their patient characteristics, to estimate PrEP success in the SHI collective in Germany as well as to determine STI diagnosis and changes of STI incidence rates before and since PrEP use. The routine data analysis methodically complements other modules of the EvE-PrEP study and is intended to be a part of an evaluation in terms of the introduction of PrEP as a benefit of health insurance in September 2019.

## Method

In order to achieve the aims raised above, routine data from three large German SHI funds (DAK Gesundheit, Techniker Krankenkasse, AOK—Die Gesundheitskasse [local health care funds]) were analyzed in anonymized form. The analyses of routine data were carried out by the Institute for Healthcare Management and Research of the University Duisburg-Essen together with the Robert Koch Institute, in cooperation with the DAK Gesundheit, Techniker Krankenkasse, and AOK Research Institute (WIdO). The Competence Center for Clinical Trials Bremen was involved as a trust center for the transmission and anonymization of the routine data.

### Subjects

Based on the reimbursement date of PrEP in the German SHI (September 1, 2019) and data availability, in order to compare the time since PrEP with time before PrEP, the transmitted routine data of insured persons of Techniker Krankenkasse and AOK—Die Gesundheitskasse concern the period January 1, 2019 to March 31, 2020. As PrEP was already voluntarily reimbursed at DAK Gesundheit from January 1, 2019, routine data from the period October 1, 2017 to March 31, 2020 were transferred. Since the coding quality for PrEP as a voluntary reimbursement did not allow the planned analysis, the transferred data period was aligned with the Techniker Krankenkasse and AOK—Die Gesundheitskasse data period for analysis. Thus, all data analyzed are from the period January 1, 2019 to March 31, 2020.

The routine data transmitted covers information regarding patient characteristics, outpatient care, inpatient care, and drugs, which are covered by health insurance.

All insured persons of the cooperating SHI with presence of prescriptions of the ATC code J05AR03 (tenofovir disoproxil and emtricitabine) within the observation period were eligible. Persons with diverse or undefined gender were excluded for data protection reasons due to the small number of cases. All persons not insured with the cooperating health insurance for more than five consecutive days in any quarter for AOK—Die Gesundheitskasse and with more than five consecutive days over the whole data period for Techniker Krankenkasse and DAK Gesundheit had been excluded. This criterion was used to ensure that no relevant diagnoses or reimbursements were missed due to insurance changes. Cases with clear evidence of HIV infection in the two quarters prior to or within the quarter of the first alleged PrEP prescription had also been excluded.

Persons with unclear age or first PrEP dispensing after the data period (some had a PrEP prescription in the data period but no dispensing, so no effects of the PrEP could be observed in this cases) were excluded from the data set and the following analyses. Additionally, persons, who never got an administration of PrEP (ATC code J05AR03) without simultaneous administration of another substance for HIV therapy/prophylaxis were excluded as well.

For the descriptive analysis of PrEP users, the age of the insured (reference date December 31, 2020), their sex, the first three digits of the postcode and their citizenship were analyzed.

To analyze adherence to daily PrEP, a quotient of the available PrEP Daily Defined Doses (DDD) prescribed and dispensed at the expense of SHI and the presumed number of days with PrEP use (first dispensing date until end of data period or until last day before presumed first HIV diagnosis (assumed end of observable PrEP use)) was calculated for each study participant. daily defined doses is a WHO concept in which the ATC codes of drugs are assigned to the usual amount of the drug prescribed for its main indication for one day. This classification is reviewed and adjusted by the Research Institute of Allgemeine Ortskrankenkasse specifically for the German context. If there were fewer days between the date of dispensing and the assumed end of observable PrEP use than the DDD, the number of DDD was reduced to the number of days from dispensing to end of observable PrEP use.

### Measures and Procedure

### PrEP Success and HIV Infections

A three-step approach was used to determine treatment success: In a first step, cases with at least one indicator of HIV infection were identified. The ICD-10 codes (in the outpatient sector, only confirmed diagnoses) and the German billing codes which were used as indicators are shown in Table 1.

**Table 1:** Indicators of HIV and STI infection

Indicators of HIV infection: ICD-10 code (including more precise multiple digits where applicable)	
B20	HIV disease resulting in infectious and parasitic diseases
B21	HIV disease resulting in malignant neoplasms
B22	HIV disease resulting in other specified diseases
B23	HIV disease resulting in other conditions
B24	Unspecified HIV disease
Z21	Asymptomatic HIV infection status
Indicators of HIV infection: German billing codes	
30920	Additional fee for the treatment of HIV-infected persons
30922	Surcharge for billing item 30920
32021	HIV infection that requires therapy
32824	HIV ribonucleic acid (RNA) testing
Examined bacterial STI: ICD-10 code (including more precise multiple digits where applicable)	
A51	Early syphilis
A52	Late syphilis
A53	Other and unspecified syphilis
A54	Gonococcal infection
A55	Chlamydial lymphogranuloma (venereum)
A56	Other sexually transmitted chlamydial diseases

In a second step, those cases were sorted out in which the indicator appears to be based on miscoding. This was assumed in three scenarios: (1) HIV ribonucleic acid (RNA) but no other indicators of HIV infection in other German billing codes or ICD-10 diagnoses or drug prescriptions, (2) after or at the same time as the HIV indication at least one PrEP prescription and no further indicator of HIV infection, (3) indicator of HIV only in a hospital diagnosis with simultaneous billing of a diagnosis-related group that explicitly excludes HIV, and subsequently no further indicator of HIV infection. In the third step, the suspected HIV cases that have not been disproven were examined on an individual basis independently by the working group of the Institute for Healthcare Management and Research of the University Duisburg-Essen and by the working group of the EvE-PrEP study at the Robert Koch Institute.

### STI Diagnosis and Changes of STI Incidence Rates Before and Since PrEP Use

In order to examine the effects of PrEP use and any associated behavioral changes on the burden of disease with regard to bacterial STI, STI rates standardized to 100 person-years were calculated at the individual level. Inpatient and confirmed outpatient diagnoses with the ICD-10 codes presented in Table 1 were taken into account. If several ICD-10 codes of the same underlying disease were coded in the same treatment case, they were counted as one disease. For this purpose, the PrEP period (since PrEP) was considered to be the time including the first day of dispensing until the assumed end of observable PrEP use (March 31, 2020 or last day before HIV infection). The comparison period (pre PrEP) is from January 1, 2019 to the day before the first PrEP dispensing.

### Statistical Analyses

Statistical analyses were performed using IBM SPSS Statistics version 27. The statistical significance of differences was tested using the Mann–Whitney U-test, the Wilcoxon test for connected samples or the Pearson chi-square test (depending on the variable). The threshold value for significance was set at *p* < 0.05. The Kolmogorov–Smirnov test was used to test for normal distribution (which in all cases led to the rejection of the *t*-test and the use of the tests listed above). We also examined the association between phase (pre PrEP versus since PrEP) and STI rates using a regression model. Due to the very high proportion of people who did not have an STI either before or since PrEP, and because the dependent variable is a count outcome, the zero-inflated negative binomial regression and the zero-inflated Poisson regression were taken into account for selection. Due to overdispersion, the zero-inflated negative binomial regression was chosen. The number of chlamydia/gonococcal/syphilis infections was included as the dependent variable in each zero-inflated negative binomial regression model. Phase (pre versus since) and, as a control, age at reference date and residence in one of the five largest cities in Germany were included as explanatory variables in both sub-models of each zero-inflated model. The logarithmised number of days in each phase was included as an offset. In addition, observations were declared independent between individuals, but not necessarily within individuals. Therefore, we used cluster adjusted standard errors with the person ID as cluster variable. The models were calculated using STATA 12.

The results regarding the estimated number of PrEP users obtained in the data set were extrapolated to the entire SHI collective in Germany adjusted for age and sex using the KM6 statistics (Federal Ministry of Health, 2019).

## Results

### Population of PrEP Users and PrEP Use

The cooperating SHI funds collectively covered about 59% of the SHI insured and almost 52% of the overall population in Germany in 2019 (Federal Ministry of Health, 2019; Federal Statistical Office of Germany [Destatis], 2023). A total of 7102 persons with PrEP use were included into the analysis. A total of 3,238,512 person-days were analyzed, including 1,029,547 person-days assumed to be with PrEP use. Table 2 shows characteristics of the included PrEP users. These were predominantly male (99%), on average 37.37 years old (standard deviation (SD) 9.58), with German citizenship (68%) and lived in large cities (primarily Berlin (43%)).

**Table 2:** Characteristics of included PrEP users between January 1, 2019 and March 31, 2020 in Germany (*n* = 7102)

Sex (frequency in %)	
Male	99.3
Female	0.7
Average age (in years; reference date December 31, 2020)	37.4
Standard deviation of age (in years)	9.6
Minimum age (in years; reference date December 31, 2020)	17.0
Maximum age (in years; reference date December 31, 2020)	75.0
Place of residence (frequency in %)	
Berlin	42.5
Cologne	6.7
Munich	6.1
Hamburg	5.5
Frankfurt am Main	3.6
Out of the five biggest cities of Germany	24.2
unknown	11.5
Most frequent citizenships	
Germany	68.2
Unknown	4.1
Italy	2.6
Spain	1.9
United States of America	1.8
Syria	1.8

Extrapolated to the entire SHI system, 11,872 PrEP users (11,789 males, 83 females) were assumed for the data period.

**Fig. 1:**
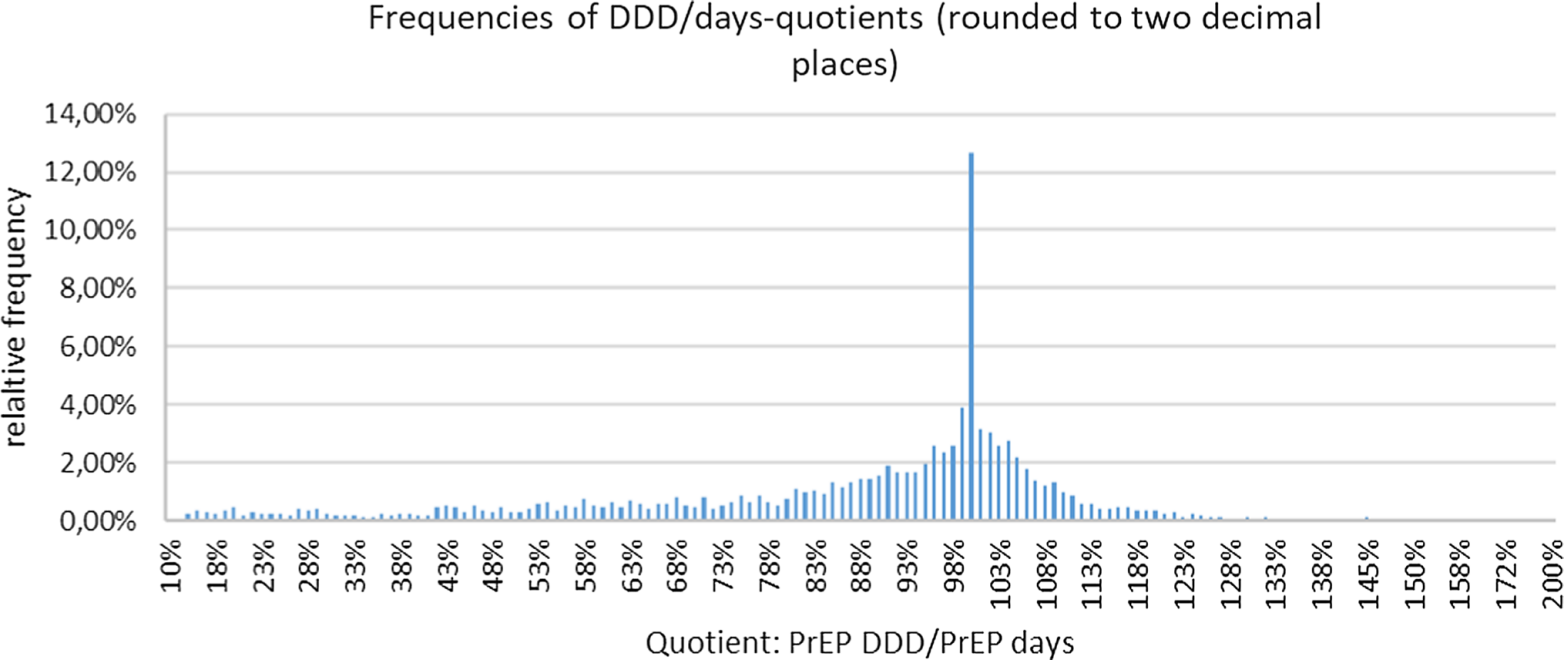
Frequencies of DDD/days quotients (rounded to two decimal places) of PrEP users between January 1, 2019 and March 31, 2020 in Germany (*n* = 7102)

Adherence to daily PrEP was expressed as the quotient of PrEP DDD and assumed days on PrEP. The average quotient was 87% (SD = 0.24), the modal value was 100%. The user with the lowest quotient had DDD for 10% of the days assumed under PrEP; while, the maximum quotient is 2, respectively, 200% (see Fig. 1). PrEP users with adherence to therapy below average were significantly younger (37.1 vs. 37.5 years) and more often from one of the biggest five cities in Germany (persons with unknown city were excluded in this sub-analysis). There was no statistically significant difference between below average users and the rest regarding their sex.

### PrEP Success and HIV Infections

In the first of the three steps described above, 384 individuals with indicators of HIV infection were identified in the data set. In 16 of these people, the indication was not classified clearly as a miscoding as described above, but only 1 of these 16 people could be identified as having a plausible sequence in the data. For this individual with a confirmable HIV diagnosis, the first pertinent billing codes were documented two days after the initial PrEP prescription and one day after the initial dispensing (German billing codes for HIV RNA testing, HIV infection that requires therapy, and additional fee for the treatment of HIV-infected persons). Thirty days after the initial PrEP prescription and 28 days after the first indicator for HIV infection, another HIV-related billing code (surcharge for additional fee for the treatment of HIV-infected persons) and the dispensing of an antiretroviral therapy [ATC code J05AR20 (emtricitabine, tenofovir alafenamide and bictegravir)] was documented. Matching observations like the continuing of the antiretroviral therapy could also be made in the further course. An HIV-ICD-10 diagnosis (B24) could not be assigned to a clear time point in the available data, but is coded for the period from Day 1 to Day 69 of the sequence described above.

### STI Diagnosis and Changes of STI Incidence Rates Before and Since PrEP Use

Table 3 shows the average normalized STI rates (normalized per 100 person-years at individual level) regarding chlamydia, gonococcal infection, and syphilis before the first dispensing of PrEP (pre PrEP) and from the first dispensing (since PrEP) to the end of the observable PrEP use. The average rate differences from pre to since PrEP were not statistically significant according to the Wilcoxon test (rates pre PrEP: chlamydia: 17.5 (95% confidence interval (CI) 16.2–18.8), gonococcal infection: 29.1 (95% CI 27.5–30.8), and syphilis: 14.6 (95% CI 13.1–16.0); rates since PrEP: chlamydia: 17.6 (95% CI 15.9–19.3), gonococcal infection: 30.7 (95% CI 28.3–33.1), and syphilis: 13.6 (95% CI 11.8–15.3)). Similarly, in the zero-inflated binomial regressions, phase (pre PrEP versus since PrEP) was not a significant coefficient for the likelihood of occurrence of the respective STI and the number of infections if there was at least one.

**Table 3:** Rates of bacterial STI of PrEP users between January 1, 2019 and March 31, 2020 in Germany (*n* = 7102)

	Rate* pre PrEP (average (95% CI))	Rate* since PrEP (average (95% CI))
Chlamydia	17.5 (16.2–18.8)	17.6 (15.9–19.3)
Gonococcal infection	29.1 (27.5–30.8)	30.7 (28.3–33.1)
Syphilis	14.6 (13.1–16.0)	13.6 (11.8–15.3)

^*^Rate normalized per 100 person-years at individual level; CI = confidence interval

**Fig. 2:**
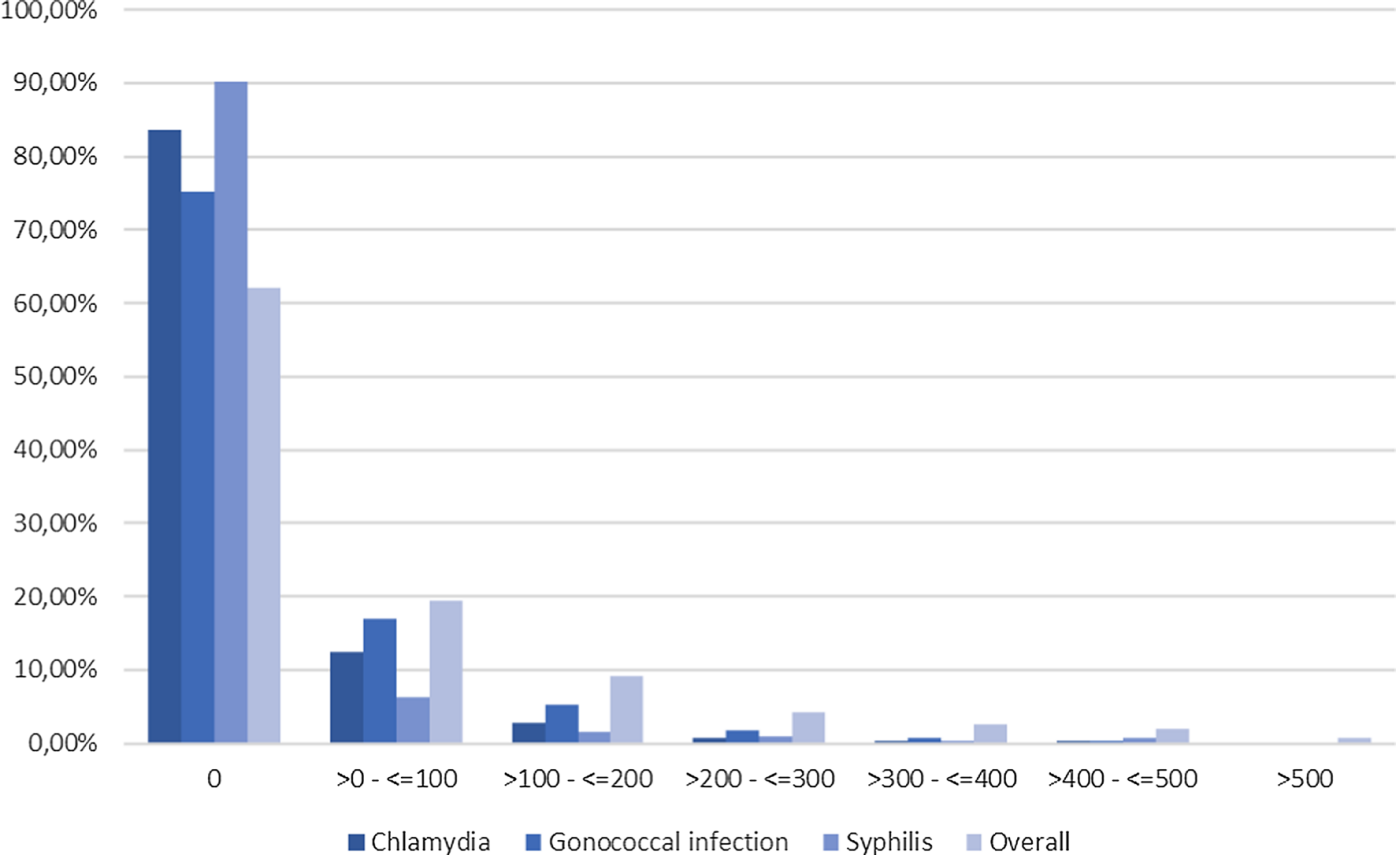
Frequencies of bacterial STI (rate normalized per 100 person-years at individual level) of PrEP users between January 1, 2019 and March 31, 2020 in Germany (*n* = 7102)

As Fig. 2 shows, the appearance of STI events is driven by a minority in the study population. Nearly two-thirds of study participants (62%) do not show any of the three diseases during the entire data period. This is particularly evident with syphilis, where 90% of study participants do not have an according diagnosis during the data period. However, compared to the average population in Germany, STI rates are considered high.

An interesting observation in addition to the primary analyses is that the data set documented 307 prescriptions that were assessed as human immunodeficiency virus post-exposure prophylaxis (PEP) prior to individual initial PrEP dispensing for 258 individuals in the data of prescriptions, but only 32 PEP prescriptions for 27 individuals emerged after initial PrEP dispensing.

## Discussion

Overall, relevant research questions regarding the effects of PrEP reimbursement in Germany could be answered on the basis of a large study population representing the PrEP user of more than half of the SHI population in Germany.

The very high proportion of men and the average age observed here are similar other recent German and international publications regarding characteristics of PrEP users (Ahaus et al., 2022; Schmidt et al., 2023; Streeck et al., 2022; Tan et al., 2021).

A high rate of PrEP users living in Berlin has been reported elsewhere. Even higher rates were observed here (Schmidt et al., 2023; Streeck et al., 2022). It has to be mentioned, that a certain degree of imprecision was unavoidable when assigning the study participants to cities, as only the first three digits of the five-digit postal codes were transmitted for data protection reasons. In addition, the demographic structure of insured persons in the participating SHI could have had an effect on the regional distribution. However, since overall, more than half of the persons with SHI in Germany were insured with the cooperating SHI, the effect is considered to be rather small.

In conclusion, the analyzed cohort matches well with other (German) PrEP cohorts in terms of patient characteristics and could confirm already published observations (Ahaus et al., 2022; Schmidt et al., 2023; Streeck et al., 2022; Tan et al., 2021). Given the high proportion of men and residents in Germany's five largest cities, this study raises the question of whether certain groups of people with a substantial HIV risk (e.g., women or people from rural areas) are currently still being inadequately reached. This is in line with findings from other independent modules of EvE-PrEP. These potential gaps in care should be the subject of further research.

Observed adherence quotients of < 100% can be interpreted in different ways. Based on the routine data, it is not possible to clarify whether this is a case of intentional on-demand use, neglect of adherence or complete discontinuation of PrEP use. The observation that those users with below-average adherence were significantly younger and live more often in large cities may suggest both, a higher prevalence of on-demand-use or a lower adherence in this collective. Quotients > 100% could result from multiple prescriptions, for example as a reserve in advance of the measures regarding the SARS-CoV-2 pandemic. It may also indicate that PrEP tablets were sometimes shared with third parties for whom presumably PrEP is not covered and who consequently did not receive adequate information, pre-diagnosis as well as accompanying follow-up testing and consultation. The average adherence to therapy reported is almost similar to the comparably calculated value determined by Schmidt et al. (2023) in another recent analysis of German PrEP care. The high level of consistency underlines the plausibility of the calculated rates, even if the rather short data period may lead to higher quotients.

The effectiveness of PrEP in preventing HIV infection has already been described nationally and internationally (Baeten et al., 2012; Fonner et al., 2016; Grant et al., 2010; McCormack et al., 2016; Molina et al., 2017; Spinner et al., 2016; Thigpen et al., 2012). Remarkable in the presented analysis is the enormously short time lag between PrEP prescription and initial HIV diagnosis in the only case with a plausible sequence. It is very likely that the HIV infection was already present before the starting of PrEP and was discovered in the course of the PrEP initiation. In other words, there is no case in the study population that cumulatively fulfills the following characteristics: non-refuted possible indicators of HIV after starting PrEP, plausible sequence in time after documented indicators of HIV, and high probability of having been infected with HIV only under PrEP use. Besides the effectiveness of PrEP, this could also be explained by the relatively short data period. Continuous monitoring of PrEP users in Germany with a longer follow-up would be of value.

In routine data, it is not possible to identify with certainty which chronologically consecutive diagnoses correspond to the same infection. Here, diagnoses of the same underlying disease which were coded in the same treatment case were counted once. Further adjustment (for example, of chronologically close diagnoses in different treatment cases) were not possible without major assumptions. While the former could lead to a small underestimation of the bacterial STI rates, the latter could lead to a small overestimation. However, basic observations such as overall high bacterial STI rates in the collective of PrEP users (with higher rates of chlamydial infections and gonorrhea than syphilis) are in line with published national and international literature (Beymer et al., 2018; Molina et al., 2017; Nguyen et al., 2018; Schmidt et al., 2023; Traeger et al., 2019). Thus, the effects described above are assumed to be minor. In addition, the focus here is on changes in rates, not on the rates itself. It was also not possible to reliably adjust for the frequency of STI tests based on the available data which may be done more routinely with and after PrEP initiation.

As mentioned above, internationally published evidence of changes regarding rates of non-HIV STI on PrEP are inconsistent (Beymer et al., 2018; Fayaz Farkhad et al., 2021; Jenness et al., 2017; McCormack et al., 2016; Molina et al., 2017; Nguyen et al., 2018; Reitsema et al., 2020; Schmidt et al., 2023; Serpa et al., 2020; Streeck et al., 2022; Traeger et al., 2019; Werner et al., 2018, 2019). The data reported here support the statement that PrEP is not necessarily associated with a statistically significant change in STI rates. Due to the end of the data period on March 31, 2020, it is not reasonable to assume that the influences of the SARS-CoV-2 pandemic obscured effects of PrEP use on STI rates. Considering the short data period, general trends in incidences of STI appear negligible as a factor in the study presented, although other studies have shown that general trends may be relevant when assessing changes in STI rates after PrEP use (McManus et al., 2020). Since the data basis is SHI routine data, PrEP use by self-payers cannot be observed. It can be assumed that some of the PrEP users paid for their PrEP themselves prior to reimbursement by the SHI, which means that days under PrEP were incorrectly added to the comparison period before PrEP. The impact of this effect on the comparison of STI rates cannot be assessed reliably.

A general limitation of routine data analyses is that they are susceptible to miscoding (Behrendt et al., 2017; Hoffmann et al., 2008; Münch et al., 2016; Schubert et al., 2010). On the one hand, routine data are nevertheless considered to be an established source of data for scientific research (Gansen, 2018; Schubert et al., 2008). On the other hand, the new reimbursement situation for PrEP may be associated with higher uncertainties regarding coding as it is the case for more established medical procedures. This is consistent with the relatively high number of indications for HIV, which could mostly be disproved and were often documented in close temporal proximity to PrEP prescriptions. Since HIV diagnoses in temporal proximity before the initial PrEP prescription led to an exclusion from the study population, it can be assumed that some PrEP users were falsely excluded from the analysis population due to miscoding. Accordingly, a slight underestimation of the number of PrEP users can be assumed. So less strict exclusion with subsequent sensitive examination is recommended for future routine data analyses of PrEP care. Other limitations resulting from the inclusion and exclusion criteria are: Persons of gender other than male or female were not included. However, this effect is considered to be minor, as their small number was the reason for privacy concerns. The exclusion of insured persons with even small absences may also have contributed to a minor underestimation of the number of PrEP users, but this approach ensured that no relevant documentation was missed.

Consistent with the above considerations, the estimate of PrEP users across the SHI system in this study is lower than another published estimate for Germany at the end of June 2020 (Marcus et al., 2022). However, in the extrapolation by Marcus et al. based on pharmacy billing data, the proportion of PrEP users without SHI and on a self-payer basis was estimated and also taken into account. This may explain some of the difference from the results presented here. A limitation in the extrapolation of the number of PrEP users from the study collective presented here to the entire SHI collective concerns the fact, that it was only possible to adjust for age and sex. Other possibly relevant characteristics (e.g., sexual orientation) were not available for this calculation. Due to the size of the sample of cooperating SHI funds, which represent more than half of the SHI insured in Germany, it nevertheless is assumed that the extrapolation is very reliable.

Specific strengths of the study presented are that the routine data are not susceptible to bias by socially desirable responses (for example, regarding treatment adherence) and that a very high number of cases could be analyzed.

### Conclusion

With access to data from three SHI funds covering a large proportion of the German population, situation after introducing PrEP as a SHI benefit could be evaluated using a large dataset. No increase in bacterial STI incidence rates, and a rather high average adherence rate was observed. Very high proportion of persons, in particular men from the largest cities in Germany among PrEP users is striking. It could be assumed that when PrEP was introduced as an SHI benefit, there were still difficulties in reaching people who are also at high risk of HIV, but who, for example, are female or live in rural areas. The long-term impact of the new reimbursement situation and research into potential barriers in access to PrEP for all relevant populations are therefore important topics for further research.

## Data Availability

Not available due to data privacy reasons.
